# NK Cell Interaction With Platelets and Myeloid Cells in the Tumor Milieu

**DOI:** 10.3389/fimmu.2020.608849

**Published:** 2020-12-23

**Authors:** Stefanie Maurer, Lucas Ferrari de Andrade

**Affiliations:** ^1^ Department of Radiology, Memorial Sloan Kettering Cancer Center, New York, NY, United States; ^2^ Precision Immunology Institute, Department of Oncological Sciences, and The Tisch Cancer Institute, Icahn School of Medicine at Mount Sinai, New York, NY, United States

**Keywords:** NK cells, platelets, myeloid cells, NKG2D, proteolytic shedding

## Abstract

Natural killer (NK) cells recognize and kill tumor cells *via* germ-line encoded receptors and polarized degranulation of cytotoxic molecules, respectively. As such, NK cells help to inhibit the development of cancers. The activating receptor NKG2D induces NK cell-mediated killing of metastasizing tumor cells by recognition of the stress-induced ligands MICA, MICB, and ULBP1-6. However, platelets enable escape from this immune surveillance mechanism by obstructing the interactions between NK cells and tumor cells or by cleaving the stress-induced ligands. It is also being increasingly appreciated that NK cells play additional roles in cancer immunity, including chemokine-mediated recruitment of antigen presenting cells in the tumor microenvironment that is followed by generation of adaptive immunity. However, the NK cell interplays with dendritic cells, and macrophages are extremely complex and involve molecular interactions *via* NKG2D and cytokine receptors. Specifically, NKG2D-mediated chronic interaction between NK cells and tumor-infiltrating macrophages causes immune suppression by differentiating NK cells toward a dysfunctional state. Here we discuss the underlying mechanisms of NK cell control by platelets and myeloid cells with focus on NKG2D and its ligands, and provide a timely perspective on how to harness these pathways with novel immunotherapeutic approaches.

## Introduction

Natural Killer (NK) cells are innate lymphocytes that recognize and kill abnormal cells, such as tumor cells and cells infected by viruses (1). NK cell activation is guided by “missing self” and “induced self” recognition of abnormal cells. “Missing self” implies that NK cells kill target cells with low/absent major histocompatibility complex (MHC) class I expression (2). “Induced self” is the expression of ligands for activating NK receptors, such as NK group 2D (NKG2D) that promotes NK cell-mediated cytotoxicity (3). NKG2D recognizes proteins upregulated by cells in response to stress, such as DNA damage, hypoxia or accumulation of unfolded proteins (4). NKG2D is present in both mice and humans, but the ligands are distinct between the two species. The human NKG2D ligands (NKG2DL) comprise MHC class I polypeptide-related sequence A (MICA), MHC class I polypeptide-related sequence B (MICB), and the six different types of UL16-binding proteins (ULBP1-6). In contrast, the murine NKG2DL are retinoic acid early inducible-1 (Rae-1) α-ϵ proteins, murine UL16-binding protein-like transcript 1 (MULT1), and H60a-c proteins (5).

NKG2D-mediated recognition of malignant cells is a major mechanism of cancer immunosurveillance (6, 7). However, as discussed in more details below, malignant cells frequently evade the recognition by NK cells via, for example, abnormal interactions with platelets. Furthermore, myeloid cells express NKG2DL in response to specific types of stimulations and NKG2D drives the interactions between NK cells and myeloid cells. Although the importance of NKG2DL on tumor cells for NK cell-driven immunity is well appreciated, how platelets and myeloid cells affect NK cell functions represent new and paradigm-shifting research fields. Here we discuss some of the recent and impactful studies that substantiate the importance of platelets and myeloid cells for effective NK cell-driven antitumor immunity.

## Platelets in-Between Tumor Cells and NK Cells

Malignant tumors frequently use the blood stream to metastasize (8). However, the blood is a hostile environment because it is highly enriched in NK cells (9). To bypass them, metastatic cells take advantage of platelets, which are small non-nuclear, megakaryocyte-derived cell fragments of the myeloid lineage that, in normal conditions, form clots to stop bleedings upon injury (10). Metastatic cells mimic injury-related clots by activating and forming hetero-aggregates with platelets. These unusual cellular clusters frequently cause thrombophlebitis, which is a medical term for inflammatory blood clots that block veins, and Trousseau’s syndrome, i.e., recurring thrombophlebitis (11). This, together with the fact that thrombocytosis (enhanced platelet count) associates with poor outcome in several solid tumor entities, suggests a causal relationship between the coagulation system and malignant dissemination (12, 13). In the following two sub-sections, we review some of the cellular and molecular mechanisms underlying the platelet-mediated immune escape of metastatic cells.

### Platelets Shield Metastatic Cells and Enable Immune Escape

Depletion of platelets directly inhibits metastases in the lungs of immunocompetent mice upon intravenous inoculation of cell lines of fibrosarcoma, melanoma, and lymphoma, whereas this effect is reversed upon additional depletion of NK cells. This indicates that platelets guard circulating tumor cells from NK cell immunosurveillance (14). Platelet activation and aggregation are mainly mediated by G-protein-coupled receptors, which upon stimulation by their respective ligands transduce intracellular signals by activating heterotrimeric G proteins (15). Platelets lacking Gαq, a subunit of heterotrimeric G proteins, are irresponsive to adenosine diphosphate, thrombin, collagen, and thromboxane, which are well known clot-forming stimuli (16). Gαq-deficient mice display substantially lower levels of metastases in the lungs after being intravenously inoculated with melanoma and lung cancer cells, and this protection was dependent on NK cells (17). Therefore, platelets contribute to dissemination of metastases. These studies predominantly utilized experimental metastasis models, with injection of tumor cells directly into the blood circulations. Further studies are needed to clarify the potential role of platelets in the context of spontaneous metastases, which also address the early steps of dissemination during tumor cell intravasation into the blood stream.

The molecular mechanisms relevant for platelet-mediated immune escape are only partially understood. Palumbo and colleagues reported that tumor cells evade NK cell-mediated surveillance *via* fibrin deposition, which enhances platelet aggregation on the tumor cell surface (17). Furthermore, the aggregated platelets transfer MHC class I molecules to tumor cells (18). MHC class I is frequently downregulated on tumor cells to evade T cell immunity, which in contrast enables recognition by NK cells *via* the ‘missing self’ mechanism (19). Surface expression of platelet-derived MHC class I complexes inhibits NK cell antitumor reactivity (20). Since platelet-derived MHC class I molecules present self-antigens, they do not induce T cell responses against metastatic cells. This intriguing mechanism of immune escape has been confirmed by a study by Placke and colleagues, who found in an *in vitro* model using shear stress that platelet-derived human leukocyte antigen A variant 2 (HLA-A*02) is transferred from platelets to tumor cells *via* trogocytosis. Therefore, platelets interfere with the “missing self” recognition of metastatic cells and dampen NK cell-driven anti-tumor immunity *via* pseudo-expression of “non-malignant” MHC class I.

Trogocytosis is frequently observed between physically interacting cells. For example, MICA/B and ULBP1-3 can be transferred from the target cell surface to NK cells at the immunological synapse (21–23). Surface molecules from antigen presenting cells are also transferred to T cells in the immunological synapses (24). Since platelets physically interact with metastasizing cells, it is possible that a plethora of other molecules with putative or confirmed roles in modulating NK reactivity can also be transferred to tumor cells in addition to MHC class I molecules (25–27).

### Platelets Promote the Shedding of NKG2D Ligands by Tumor Cells

**Figure 1:**
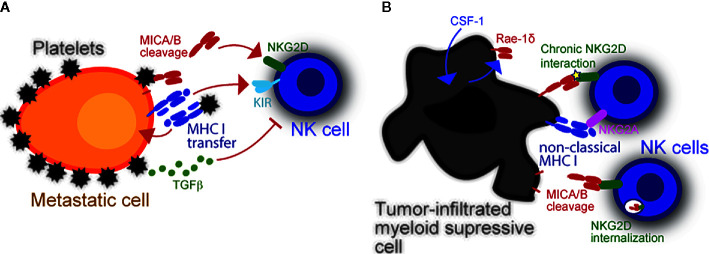
Modulation of NK cell reactivity by platelets and myeloid cells. **(A)** Platelets obstruct NK cells and enable escape of metastasizing tumor cells. Platelets also provide specific immune modulatory molecules like MHC class I which inhibits NK cells. The latter can be transferred into the tumor cell membrane *via* trogocytosis to inhibit missing self-driven NK cell cytotoxicity. Their cognate Killer-cell immunoglobulin-like receptors (KIRs) inhibit NK antitumor responses upon stimulation. Platelets can also dampen induced self recognition of tumor cells *via* NKG2D. Platelet-derived metalloproteases (i.e., ADAM10 and ADAM17) cleave NKG2DL from the tumor cell surface. Platelet-released TGF-β also causes NKG2D downregulation, thereby further hindering NK cell antitumor response. **(B)** The expression of NKG2DL is not restricted to malignant cells. In fact, DC and macrophages can also express NKG2DL upon stimulation or infection, which in-turn induces NK cells to proliferate and produce interferon-γ (IFNG). Virus-infected myeloid cells may become targets and be killed by NK cells upon NKG2D recognition. Intratumoral myeloid cells also express NKG2DL. It is currently unknown what induces NKG2DL expression in intratumoral myeloid cells, yet a potential mechanism is *via* cellular stress inside the hypoxic tumor microenvironment. These cells benefit tumors by inhibiting NK cells *via* chronic NKG2D interaction with low affinity ligands, which cause NKG2D internalization.

High levels of NKG2DL tip the balance toward NK cell activation (28, 29). However, certain ligands are subjected to proteolytic cleavage, which interferes with NKG2D recognition. It is well known that tumor cells cleave their own NKG2DL *via* expression of ‘a disintegrin and metalloproteinase domain-containing protein’ (ADAM) 10 and ADAM17 (5, 30, 31). Interestingly, recent studies also suggested platelet-mediated cleavage of NKG2DL since platelets express both proteases (32, 33) that mediate NKG2DL shedding on tumor cells (34–37). We recently discovered that tumor cell-associated NKG2DL, predominantly MICA and MICB, were cleaved following interaction with platelets or platelet releasate. We also demonstrate that platelet-mediated shedding of NKG2DL dampens NK cell antitumor immunity by reducing the activating signals. Of note, expressions of both proteolytic enzymes are increased on platelets from patients with non-small cell lung cancer, thus suggesting that cancer patients-derived platelets have enhanced proteolytic cleavage capacity (38). Furthermore, platelets express NKG2DL, in particular ULBP2, which may be released as soluble form (39). The biological activity of soluble ULBP2 is not well known, but ULBP2 shedding may also inhibit recognition of platelet-tumor aggregates by NKG2D. Altogether, platelets modulate the expression and release of NKG2DL and thereby inhibit NKG2D-mediated NK cell recognition of abnormal cells (**Figure 1A**).

Platelets also inhibit NK cells by inducing the NKG2D, downregulation, thus hindering “induced self” recognition. Upon stimulation with agonists or interaction with tumor cells, platelets release a variety of factors, the collectivity of which is herein referred to as “releasate”. Platelet releasate includes large amounts of active transforming growth factor β (TGF-β) (40). TGF-β impairs NK effector function by downregulating NKG2D, as determined with samples from cancer patients (41–43). Salih and colleagues have also demonstrated that TGF-β is released by platelets upon interaction with tumor cells, and it impairs NK cell cytotoxicity and production of interferon-γ *via* NKG2D downregulation (44). Others reported that TGF-β directly inhibits expression of NKG2DL on solid tumors (45, 46). Whether this is also true for platelet-derived TGF-β is yet to be shown. Altogether, platelets promote dissemination of metastases by obstructing NK cells and producing immunosuppressive molecules (**Figure 1A**).

## The Interaction Between NK Cells and Myeloid Cells

The antitumor immunity is a coordinated interplay of multiple leukocyte populations, including NK cells and myeloid cells (e.g. dendritic cells, macrophages, etc) (47). NK cells can recruit (see below) and interact with myeloid cells *via* chemokines and cytokines, respectively. For example, interleukin-15 is a well-known cytokine produced by dendritic cells (DC) that stimulates NK cells (48). Interestingly, myeloid cells can also express NKG2DL, but they are not killed by NK cells. As discussed below, the NKG2D-driven interplays between NK cells and myeloid cells cause profound impacts on the antitumor immunity by dictating the functionality of NK cells. Here we review some of the key and paradigm shifting studies that revealed several mechanisms of interactions between NK cells and myeloid cells.

### NK Cell-Mediated Recruitment of Dendritic Cells Into Tumors

The effector functions of NK cells are not restricted to cytotoxicity. As revealed by gene expression analyses, specialized NK cell populations infiltrate into melanoma metastases. Although blood and tumor-infiltrating NK cells express predominantly perforin and granzyme genes that are related to cytotoxicity, tumor-infiltrating NK cells upregulate *XCL1*, *XCL2*, *CCL3*, *CCL4*, *CCL4L2*, and *CCL5* that are chemokine genes. Tumor-infiltrating NK cells differentiate into two main populations with distinct profiles of chemokine gene expression: *XCL1* ^+^ *XCL2* ^+^ NK cells and *CCL3* ^+^ *CCL4* ^+^ *CCL4L2* ^+^ *CCL5* ^+^ NK cells. The first NK cell population also upregulates *TIGIT*, an inhibitory receptor, whereas the later one also upregulates *IL7R*, a cytokine receptor gene (49).

Conventional type-1 dendritic cells (cDC1) express XCR1, which is the receptor for XCL1. This DC population takes up particles of dead tumor cells, migrates to draining lymph nodes, and presents tumor cell-derived antigens to CD8^+^ T cells (50). The XCR1 – XCL1 axis promotes recruitment of cDC1 into tumors and promotes adaptive immunity against cancers. CCL5 also contributes to cDC1 recruitment into tumors. In a mouse BRAF^V600E^-mutant melanoma model, intratumoral NK cells upregulate *Xcl1* and *Ccl5* whereas NK cell depletion lowered the numbers of tumor-infiltrating cDC1. Antibody-mediated blockade of XCL1 and CCL5 also inhibited tumor-infiltrating cDC1, thus leading to the conclusion that intratumoral NK cells produce XCL1 and CCL5 which in turn recruit cDC1 (51). Another study also showed that NK cell depletion inhibits the cDC1 infiltration into mouse B16F10 melanoma tumors, whereas melanoma patients who responded to checkpoint blockade immunotherapy have higher levels of intratumoral NK cells (52). Therefore, the NK cell-mediated recruitment of cDC1 is an additional role, beyond cytotoxicity, played by NK cells in the antitumor immunity.

### The Interaction Between NK Cells and NKG2DL^+^ Dendritic Cells Has Two Distinct Outcomes: Activation or Inhibition

Myeloid cells express NKG2DL in response to certain stimuli. For example, interferon-α triggers MICA expression on human monocyte-derived DC, which in turn promotes NK cell-mediated cytotoxicity against K562 myeloid leukemia cells *in vitro*. A MICA antibody that blocks interaction with NKG2D consequently inhibits DC stimulation of NK cells (53). LPS, Poly I:C, and virus infections (e.g. measles virus, influenza virus) also trigger the expression of several ULBP molecules and MICB on DC. Virus-infected DC promote, in a NKG2D-dependent manner, NK cell proliferation and trigger interferon-γ production (54). However, the NKG2D-driven crosstalk with DC does not always benefit NK cells. To study the interaction between NK cells and DC *via* NKG2D *in vivo*, Morvan et al. developed a mouse strain in which Rae-1ϵ, a murine NKG2DL, is constitutively expressed by CD11c^+^ DC. Although the numbers of NK cells are apparently normal in these mice, these NK cells downregulate NKG2D. NK cells from CD11c-Rae-1ϵ mice allowed the expansion of Rae-1ϵ -expressing splenocytes in a model of NKG2D-dependent lysis of target cells *in vivo* (55). Therefore, NKG2D-driven chronic interaction with DC inhibits NK cells whereas the expression of NKG2DL on DC in response to acute infection and interferon-α promotes NK cell functions.

### NK Cells Downregulate NKG2D Upon Interaction With NKG2DL+ Macrophages

Macrophages also express Rae-1α, Rae-1β, and Rae-1γ upon treatment with LPS, poly I:C, or E. coli. Rae-1δ and Rae-1ϵ were also expressed by murine macrophages that were treated with LPS, but were not expressed by macrophages from *Myd88^-/-^* mice. MyD88 is an adaptor molecule for toll like receptors (TLR), thus suggesting that the upregulation of NKG2DL by macrophages is downstream of TLR signaling. Of note, NK cells co-cultured with Rae-1^+^ macrophages downregulate the surface expression of NKG2D (56). However, macrophages upregulate Qa-1 to protect themselves from NK cell-mediated attack. Qa-1 binds to the NK group 2A receptor, which inhibits NK cells (57). Furthermore, intratumoral macrophages also express Rae-1δ in response to tumor-derived colony-stimulating factor-1, and Rae-1δ^+^ macrophages caused NKG2D downregulation upon co-culture with NK cells (58).

Human macrophages upregulate MICA upon treatment with LPS or CL097, which are TLR4 and TLR7/8 agonists, respectively. CL097 also triggers the expression of MICB (59). Even monocytes express MICA in response to LPS or poly I:C. These MICA^+^ monocytes are not lysed by NK cells, but promote the interferon-γ production. A MICA antibody partially inhibited the interferon-γ production by NK cells; this effect was partial likely because LPS also triggers the expression of interleukin-12 that potently promotes interferon-γ production by NK cells. The MICA^+^ monocytes also caused a modest NKG2D downregulation on the surface of NK cells (60). However, an independent study did not observe NKG2D downregulation on NK cells that were co-cultured with LPS-treated monocytes, macrophages, or DC. This work confirmed that LPS triggers MICA expression in macrophages and found that these cells also express ULBP3. NK cells lysed the LPS-treated macrophages in a NKG2D-dependent manner (61).

MULT1 is an intriguing NKG2DL that has unique properties. A recent study showed that B16F10 melanoma cells engineered to secrete a truncated MULT1 protein, which lacks the transmembrane domain, form small subcutaneous tumors following inoculation into C57BL/6 mice. NKG2D was upregulated on intratumoral NK cells, whereas NK cell depletion with anti-NK1.1 enabled the growth of B16F10 tumors secreting MULT1. Soluble MULT1 interfered with NKG2D – Rae-1 interactions between intratumoral NK cells and myeloid cells, likely because MULT1 has higher affinity to NKG2D compared to Rae-1. Therefore, soluble MULT1 displaces the NKG2D – Rae-1 chronic interaction and consequently restores NK cells (62).

NKG2D downregulation upon chronic interaction was observed in several of the studies cited above and is frequently assumed to represent a mechanism of immune suppression. However, NKG2D is also internalized upon ligand interaction at the immunological synapse between NK cells and myeloid cells. This enables physical approximation between NKG2D and its associated intracellular signaling pathways that trigger NK cell-mediated cytotoxicity (63). Therefore, NKG2D can be downregulated following the interactions between NK cells and myeloid cells also to enable intracellular signaling, which will ultimately dictate the NK cell functionality.

### Myeloid Cells Shed MICA and MICB

The proteolytic shedding of MICA and MICB by tumor cells causes immune escape *via* downregulation of these NKG2DL (30, 35, 36, 64, 65). The shedding of MICA and MICB are multi-step processes that start with the removal of disulfide bonds in the MICA and MICB alpha-3 (α3) domains by disulfide isomerases followed by cleavage by metalloproteases (64, 66). In contrast, monoclonal antibodies against the α3-domain inhibit the MICA and MICB shedding, while enabling NKG2D recognition and triggering antibody-dependent cellular cytotoxicity by NK cells. These antibodies inhibit the outgrowth of syngeneic melanoma in immunocompetent mice and human melanoma in NSG mice reconstituted with human NK cells (67). Even tumors with mutations associated with resistance to T cell checkpoint blockade were effectively treated by these α3-domain-specific antibodies (68). Of note, macrophages treated with acetylated low-density lipoproteins, an *in vitro* model of foam cells present in atherosclerotic lesions, upregulate MICA and MICB expression (69). The α3-domain-specific antibodies stabilized MICA and MICB on the surface of these macrophages (67). Therefore, macrophages, like tumor cells, proteolytically shed MICA and MICB.

In summary, NKG2D mediates the interplay between NK cells and myeloid cells. Myeloid cells upregulate NKG2DL in response to acute infection or detection of pathogen-associated molecular patterns, which in turn stimulate NK cells *via* NKG2D (**Figure 1B**). In contrast, intratumoral myeloid cells also upregulate NKG2DL in response to unknown stimuli, but NKG2DL^+^ myeloid cells inhibit intratumoral NK cells. Macrophages shed MICA and MICB, but how MICA and MICB shedding by macrophages influences NK cells remains unknown. Therefore, the NKG2D-driven interaction between NK cells and myeloid cells is an intriguing research area that challenges current paradigms and offers opportunities to develop therapeutic approaches.

## Conclusion and Perspective

NK cells are being increasingly appreciated and exploited by new immunotherapeutic modalities for cancers. New insights about how platelets and myeloid cells affect NK cells may help to develop cancer immunotherapies. We envision that platelets and myeloid cells can be harnessed to enable NK cell recognition of metastasizing cells and promote NK cell functions in the tumor environment, respectively, with novel therapeutic approaches. This may include targeting immune checkpoints involved in tumor cell - platelet - NK cell interaction or by already approved drugs (70). Beyond that, adaptive immunity may be promoted by mimicking the NK cell-mediated recruitment of cDC1s into tumors *via* local inoculation of XCL1 and CCL5. We here reviewed current knowledge on the cellular interplays of platelets - myeloid and NK cells with a focus on the NKG2D/NKG2DL system and provided a systematic overview on the rationale to target this axis, which may include but is not limited to prevention of NKG2DL shedding by a blocking antibody. While therapeutic targeting of these axes in the tumor microenvironment appears to be promising, further investigations are warranted to study the pathophysiologic role of the here summarized mechanisms in the context of different tissues, tumor entities and disease stages.

## Author Contributions

Manuscript writing and editing were jointly done by SM and LFdA. All authors contributed to the article and approved the submitted version.

## Funding

SM is supported by the Deutsche Forschungsgemeinschaft, MA 8774/1-1. LFdA is supported by seed funds provided by the Icahn School of Medicine at Mount Sinai.

## Conflict of Interest

LFdA is co-inventor in an issued patent about an alpha-3 domain-specific antibody and serves as consultant for Cullinan Oncology.

The remaining author declares that the research was conducted in the absence of any commercial or financial relationships that could be construed as a potential conflict of interest.
